# Alterations of DNA methylation were associated with the rapid growth of cortisol-producing adrenocortical adenoma during pregnancy

**DOI:** 10.1186/s13148-021-01205-3

**Published:** 2021-12-04

**Authors:** Chuan Wang, Yujing Sun, Xiaofei Yin, Ruoqi Feng, Ruiying Feng, Mingyue Xu, Kai Liang, Ruxing Zhao, Gangli Gu, Xuewen Jiang, Peng Su, Xiaofang Zhang, Jinbo Liu

**Affiliations:** 1 Department of Endocrinology, Qilu Hospital, Cheeloo College of Medicine, Shandong University, Jinan, 250012 People’s Republic of China; 2 Institute of Endocrine and Metabolic Diseases, Shandong University, Jinan, 250012 Shandong Province People’s Republic of China; 3 Jinan Clinical Research Center for Endocrine and Metabolic Diseases, Jinan, 250012 Shandong Province People’s Republic of China; 4 Department of Urology, Qilu Hospital of Shandong University, Jinan, 250012 People’s Republic of China; 5 Department of Pathology, Qilu Hospital of Shandong University, Jinan, 250012 People’s Republic of China

**Keywords:** DNA methylation, Cushing’s syndrome, Pregnancy, Cortisol-producing adrenocortical adenoma

## Abstract

**Background:**

Cortisol-producing adrenocortical adenoma (CPA) during pregnancy rarely occurs in clinic. Growing evidence suggests that DNA methylation plays a key role in adrenocortical adenomas. The present study aims to examine the genome-wide DNA methylation profiles and identify the differences in DNA methylation signatures of non-pregnant and pregnant patients with CPA.

**Results:**

Four pregnant and twelve non-pregnant patients with CPA were enrolled. The pregnant patients with CPA had higher serum cortisol, Estradiol, Progesterone, and human chorionic gonadotropin concentration, while having lower serum FSH (follicle-stimulating hormone) and luteinizing hormone concentrations (*P* < 0.01). Compared with the non-pregnant patients, the duration is shorter, and the growth rate of the tumor is faster in pregnant patients with CPA (*P* < 0.05). Morphology and cell proliferation assay showed that the percentage of Ki-67 positive cells in CPA were higher in pregnant group than non-pregnant group (8.0% vs 5.5%, *P* < 0.05). The DNA methylation analysis showed that Genome-wide DNA methylation signature difference between pregnant and non-pregnant with CPA, that the pregnant group had more hypermethylated DMPs (67.94% vs 22.16%) and less hypomethylated DMPs (32.93% vs 77.84%). The proportion of hypermethylated DMPs was relatively high on chromosomes 1 (9.68% vs 8.67%) and X (4.99% vs 3.35%) but lower on chromosome 2(7.98% vs 12.92%). In pregnant patients with CPA, 576 hypomethylated DMPs and 1109 hypermethylated DMPs were identified in the DNA promoter region. Bioinformatics analysis indicated that the Wnt/β-Catenin pathway, Ras/MAPK Pathway and PI3K-AKT Pathway were associated with the development of CPA during pregnancy.

**Conclusions:**

Genome-wide DNA methylation profiling of CPA in non-pregnant and pregnant patients was identified in the present study. Alterations of DNA methylation were associated with the pathogenesis and exacerbation of CPA during pregnancy.

**Supplementary Information:**

The online version contains supplementary material available at 10.1186/s13148-021-01205-3.

## Background

Cushing’s syndrome (CS) during pregnancy is a rare endocrine disorder, with only about 220 cases reported worldwide [1]. CS is usually associated with severe maternal and fetal complications. Fetal mortality in pregnant women with CS is as high as 25–40% due to spontaneous abortion, stillbirth, and prematurity [2, 3].

In non-pregnant patients with CS, adrenocorticotropic hormone (ACTH)-secreting pituitary tumors account for 60–70% of cases [2]. The majority of CS during pregnancy, however, is caused by cortisol-producing adrenocortical adenoma (CPA) [3]. Surprisingly, some cases of CS during pregnancy resolve spontaneously after delivery [4]. It was previously suggested that the hormonal metabolic milieu during pregnancy may trigger the development of CPA. Most studies indicated that an aberrant expression of luteinizing hormone (LH)/human chorionic gonadotropin (hCG) receptor (LHCGR) in CPA played a critical role in the pathogenesis and exacerbation of CPA during pregnancy [5, 6]. However, cortisol hypersecretion was not observed in all pregnant women [7]. Furthermore, some cases of LHCGR-positive adrenal tumors or macronodular hyperplasia did not respond to hCG or LH [8]. As a result, alternative pathogenic mechanisms of CPA during pregnancy were indicated.

DNA methylation is a fundamental epigenetic mechanism that regulates gene expression. Gene transcription is generally active in unmethylated DNA regions, and DNA methylation reduces gene expression [9]. Recent research found that excess hormone caused by an adrenocortical hormone-producing adenoma was linked to DNA methylation. Cytochrome P450 family 11 subfamily B number 1 (CYP11B1) was upregulated in CPA by DNA hypomethylation of its promoter region [4, 9]. Given the significant changes in hormones and internal environment that occurred during pregnancy, we hypothesized that the alterations of DNA methylation might be involved in the development of CPA in patients during pregnancy.

In this study, we collected clinical data and examined the genome-wide DNA methylation profiles of non-pregnant and pregnant patients with CPA. We aimed to identify the differences in DNA methylation signatures of non-pregnant and pregnant patients with CPA, which might be associated with the pathogenesis and exacerbation of CPA during pregnancy.

## Results

### Clinical manifestation

**Fig. 1:**
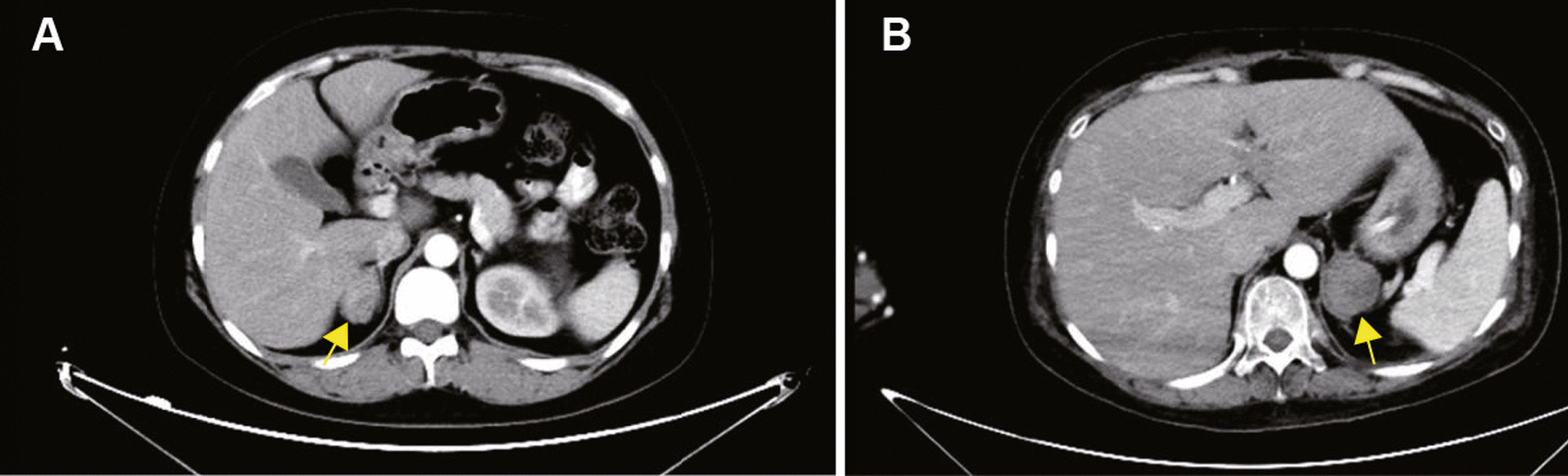
Representative adrenal CT images of non-pregnant and pregnant patients. **A** Adrenal CT image of non-pregnant patient with CPA. **B** Adrenal CT image of pregnant patient with CPA. The adenoma was marked by yellow arrow

Clinical characteristics of CPA patients with or without pregnancy showed in Table 1. No significant difference in age, BMI, SBP, DBP, potassium and tumor size was found between the non-pregnant and pregnant group (*P* > 0.05). The pregnant patients with CPA had higher serum cortisol, E2, Progesterone, and HCG concentration, while having lower serum FSH and LH concentrations (*P* < 0.01). Furthermore, compared with the non-pregnant patients, the duration is shorter, and the growth rate of the tumor is faster in pregnant patients with CPA (*P* < 0.05). The CT imaging of CPA was shown in Fig. 1.

**Table 1:** Clinical characteristics of CPA patients with or without pregnancy

Characteristics	CPA without pregnancy (*n* = 12)	CPA with pregnancy (*n* = 4)	*P* value
Age (years)	30.08 ± 3.11	28.50 ± 2.33	0.783
BMI (kg/m^2^)	24.90 ± 1.14	27.68 ± 0.69	0.197
SBP (mmHg)	149.83 ± 5.01	152.50 ± 3.66	0.773
DBP (mmHg)	102.00 ± 2.87	97.00 ± 3.08	0.365
Duration of disease (months)	25.92 ± 5.81	4.25 ± 0.85	0.003
K (mmol/L)	3.63 ± 0.15	2.93 ± 0.41	0.057
FSH (IU/L)	5.14 ± 0.49	0.13 ± 0.02	< 0.001
LH (IU/L)	4.00 ± 0.46	0.19 ± 0.03	< 0.001
E2 (pmol/L)	296.48 ± 65.25	7446.43 ± 610.97	0.001
PRL (nmol/L)	86.42 ± 6.49	192.30 ± 48.13	0.002
P (nmol/L)	1.11 ± 0.50	161.70 ± 5.58	< 0.001
HCG (IU/L)	0.27 ± 0.09	45,658.00 ± 9511.54	< 0.001
Cortisol (nmol/L) (8 am)	593.06 ± 51.29	975.35 ± 140.51	0.006
Cortisol (nmol/L) (4 pm)	577.95 ± 44.19	984.74 ± 150.99	< 0.001
Cortisol (nmol/L) (12 pm)	554.11 ± 60.96	935.13 ± 81.89	0.006
ACTH (pmol/L) (8 am)	< 1.00	< 1.00	–
ACTH (pmol/L) (4 pm)	< 1.00	< 1.00	–
ACTH (pmol/L) (12 pm)	< 1.00	< 1.00	–
Tumor size (cm^2^)	10.31 ± 1.01	10.69 ± 0.80	0.830
Growth rate of tumor (m^2^/month)	1.00 ± 0.33	2.86 ± 0.57	0.012

Data are mean ± SEM

BMI, Body Mass Index; SBP, Systolic blood pressure; DBP, diastolic blood pressure; K, potassium; FSH, Follicle-stimulating hormone; LH, luteinizing hormone; E2, Estradiol; PRL, prolactin; P, progesterone; HCG, human chorionic gonadotropin; ACTH, adrenocorticotrophic hormone

### Cell morphology

**Fig. 2:**
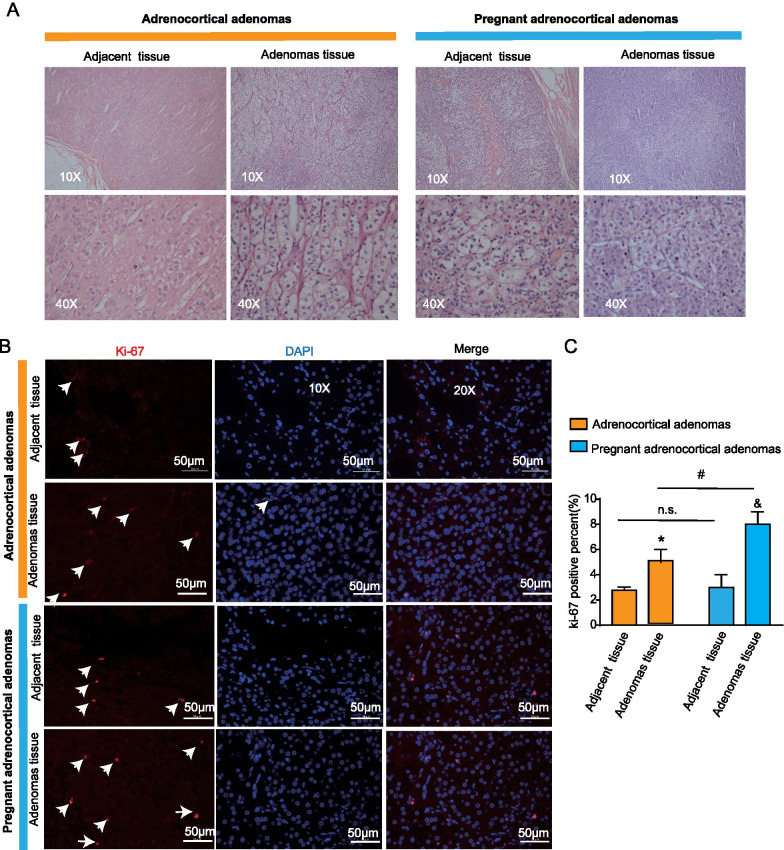
Morphologic characteristics of adrenocortical adenomas of pregnant and non-pregnant groups. **A** Hematoxylin and eosin staining (HE) of adrenocortical adenomas in pregnant and non-pregnant patients (magnification 10×, 20×, or 40×). **B** Representative images of the cell proliferation marker Ki-67 immunopositivity staining of the two groups (Scale bar: 50 μm). **C** Statistical analysis of figure (**B**)

Morphology is a critical and fundamental step in the diagnosis of adrenocortical adenoma. The histology results revealed that the adenoma cells were enlarged, and cytoplasmic lipids were increased, with lipid-rich foamy cytoplasm (Fig. 2A). Ki-67 staining was used to assess tumor cell proliferation activity. It showed that the percentage of Ki-67 positive cells are higher in the pregnant group than the non-pregnant group (8% vs 5.5%, *P* < 0.05) (Fig. 2B, C).

### Comparative analysis of the genome-wide DNA methylation status between the adjacent tissue and adenoma tissue of the non-pregnant patients with CPA

**Fig. 3:**
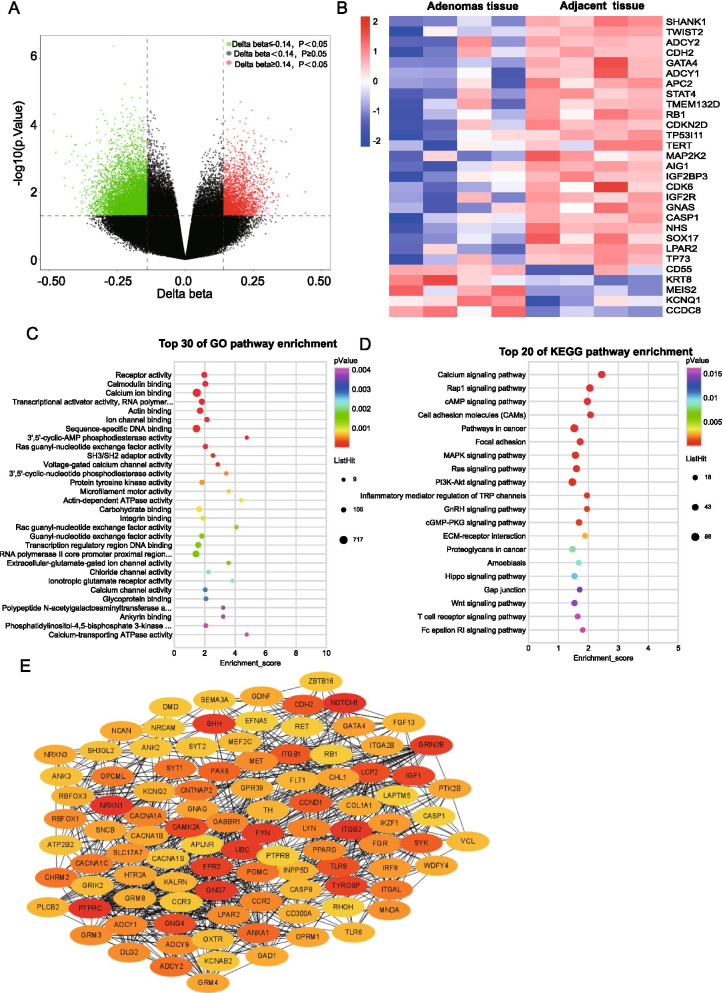
DNA methylation and functional analysis of the overall region between the adjacent tissue and adenoma tissue of the non-pregnant patients with CPA. **A** Volcano graph illustrating the difference in DNA methylation across the entire chromatin region between the adjacent tissue and adenoma tissue of the non-pregnant patients with CPA. **B** The heatmap of DNA methylation differences in the overall region stratified by hyper- and hypo-DMPs that previously reported. **C**, **D** Enriched GO (Top 30) and KEGG pathways (Top 20) in hypo-DMPs associated with DNA hypomethylation genes in the adenoma tissue of the non-pregnant patients. **E** PPI and functional enrichment network of the overall region hypomethylated DMPs associated genes in the adenoma tissue of the non-pregnant patients using the STRING and Cytoscape software

To determine whether our present study was consistent with previous research in DNA methylation of CPA. Global DNA methylation was measured by Illumina's Infinium human methylation 850 K BeadChip. The differential methylation positions (DMPs) between adjacent and adenoma tissues were shown on the volcano plot (Δ*β* ≤  − 0.14 or Δ*β* ≥ 0.14, *P* < 0.05). Our results found that 10,053 DMP sites differed between adenomas and adjacent tissues, containing 2229 hypermethylated and 7824 hypomethylated sites in non-pregnant patients with CPA (Fig. 3A). A heat map was used to show the gene methylation level signature (Fig. 3B). According to previous studies, we found five hypermethylated genes and twenty-four hypomethylated genes differed between adenomas and adjacent tissues in our results that had been reported previously and we listed the studies in Additional file 1.

To identify the genome-wide DMPs difference between adenomas and adjacent tissues of non-pregnant adrenocortical adenomas groups, the GO and KEGG pathway enrichment was analyzed. For GO term analysis, we chose 7824 DMPs (containing 2925 genes) with hypomethylation in the adenoma tissue than the adjacent tissue. We just show the date that involved in molecular function. We found that adenoma tissues had the same hypo-methylated driven genes involved in adrenocortical adenoma development signaling, such as calcium ion binding, 3′,5′-cyclic-AMP phosphodiesterase activity, SH3/SH2 adaptor activity (Fig. 3C and Additional file 2). To determine the potential function differences between the two groups, KEGG pathways were performed on the hypo-methylated genes in adenoma tissues, which showed that Calcium signaling pathway, Cell adhesion molecules (CAMs), cAMP signaling pathway, MAPK signaling pathway, PI3K-Akt signaling pathway, WNT signaling and positive regulation of antigen receptor-mediated signaling pathway (Fig. 3D and Additional file 3). These pathways play important roles in the development of adrenocortical adenomas.

We then used the STRING-database software and Cytoscape software to analyze the top 1800 genes. It displays the number of nodes at 1203 and the number of edges showing 5807, then we analyze the functional enrichment in the network. It was considered that the top 20 genes with high degree of connectivity as the hub genes of PTPRC, SHH, CAMK2A (calcium ion binding), NOTCH1, ANXA1(transcription factor activity), NRXN1 (receptor activity), ITGB2(cell adhesion molecule binding), GNG7, GNG4 (signal transducer activity), FYN (phosphatidylinositol-4,5-bisphosphate 3-kinase activity), GRIN2B (extracellular-glutamate-gated ion channel activity), TYROBP (receptor binding), IGF1 (growth factor activity), TLR8V (drug binding), ITGB1 (cell adhesion molecule binding), CDH2 (beta-catenin binding), CCND1 (transcription factor binding), SYK (protein tyrosine kinase activity), PAX6 (HMG box domain binding), ADCY2 (phosphorus-oxygen lyase activity) in term of molecular function(Fig. 3E and Additional file 4).

### The difference of genome-wide DNA methylation profiles between pregnant and non-pregnant patients with CPA

**Fig. 4:**
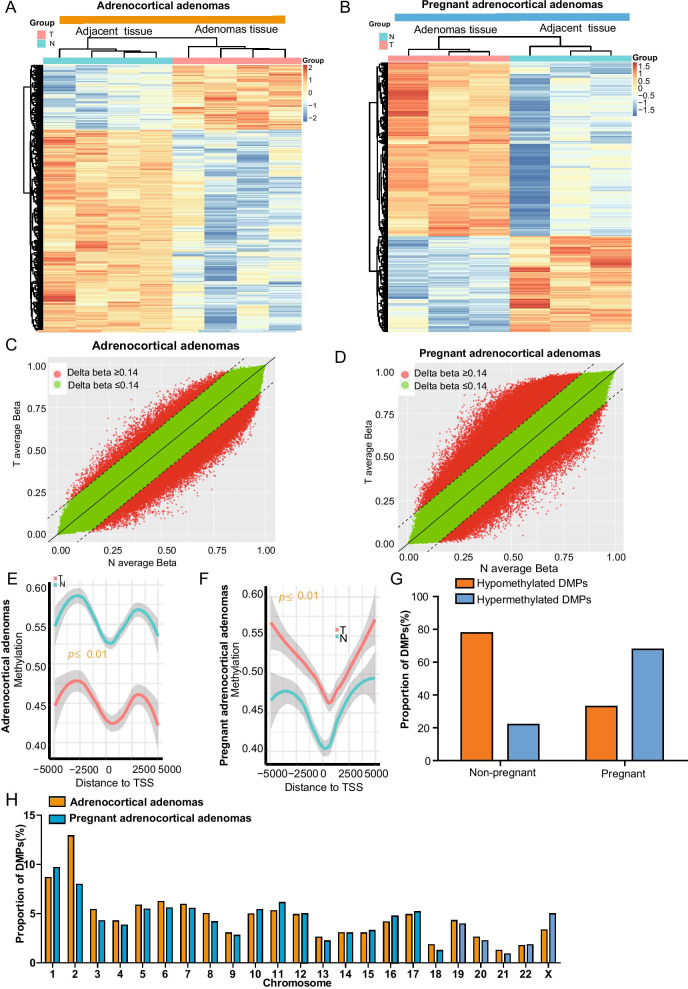
Genome-wide DNA methylation signature difference between pregnant and non-pregnant adrenocortical adenomas. **A**, **B** Heatmap of DNA methylation in adrenocortical adenomas in non-pregnant (**A**) and pregnant (**B**) patients based on genome-wide DNA methylation status. Heat maps are used to compare the similarity and difference of the two groups. For drawing the figures, top 5000 differences DMPs were chosen; if the differences DMPs sites were less than 5000, all differences will be displayed. The results of the heatmap drawn directly using the value shown on the left side of the figure. **C**, **D** Scatter plots are used to show the difference in genome-wide DNA methylation between the C and D groups. The red points denote points mean Δ*β* > 0.2, and the green points denote points mean Δ*β* < 0.2. **E**, **F** TSS methylation distribution map of non-pregnant (**E**) and pregnant groups (**F**). We extracted the relevant information from differential methylation sites and compared the methylation differences between groups of 5 kb upstream and downstream of the transcription start site TSS. **G** The genome-wide DNA distribution of hypermethylated and hypomethylated DMPs in non-pregnant and pregnant groups. **H** The chromosomal distribution of methylated DMPs in non-pregnant (orange) and pregnant (blue) groups

To identify the genome-wide DNA methylation difference between pregnant and non-pregnant groups, hierarchical clustering was used to analysis the methylation patterns of DMPs. The top 5000 DMPs differences (*p* < 0.05) were chosen to draw the heatmap and scatter plot figures. The results showed that the two groups have distinct methylation patterns. The pregnant group contained higher hypermethylated DMPs (5043 vs 2229 sites) and lower hypomethylated (2477 vs 7824 sites) (Fig. 4A–D). We also extracted the relevant information from the genome-wide DNA methylation sites and examined the differences between the two groups of 5 kb upstream and downstream of the transcription start site (TSS). The TSS upstream and downstream methylation distribution results visually showed that the chromosomal hyper-methylation sites regions of the pregnant (Fig. 4F) are higher than those of non- pregnant group (Fig. 4E). To further investigate the chromosomal distribution of the DNA methylation status corresponding to the 5000 DMPs, we then determined the proportion of DMPs sites that were hypermethylated and hypomethylated, respectively, on pregnant and non-pregnant chromosomes (Fig. 4G). The results also show that the pregnant groups had more hypermethylated DMPs (67.94% vs 22.16%) and less hypomethylated DMPs (32.93% vs 77.84%). We calculated the proportion of DNA methylation status on each chromosome, the proportion of hypermethylated DMPs was higher on chromosomes 1 and X but lower on chromosome 2 (Fig. 4H).

### Identifying the promoter DNA methylation region signature of CPA during pregnancy

**Fig. 5:**
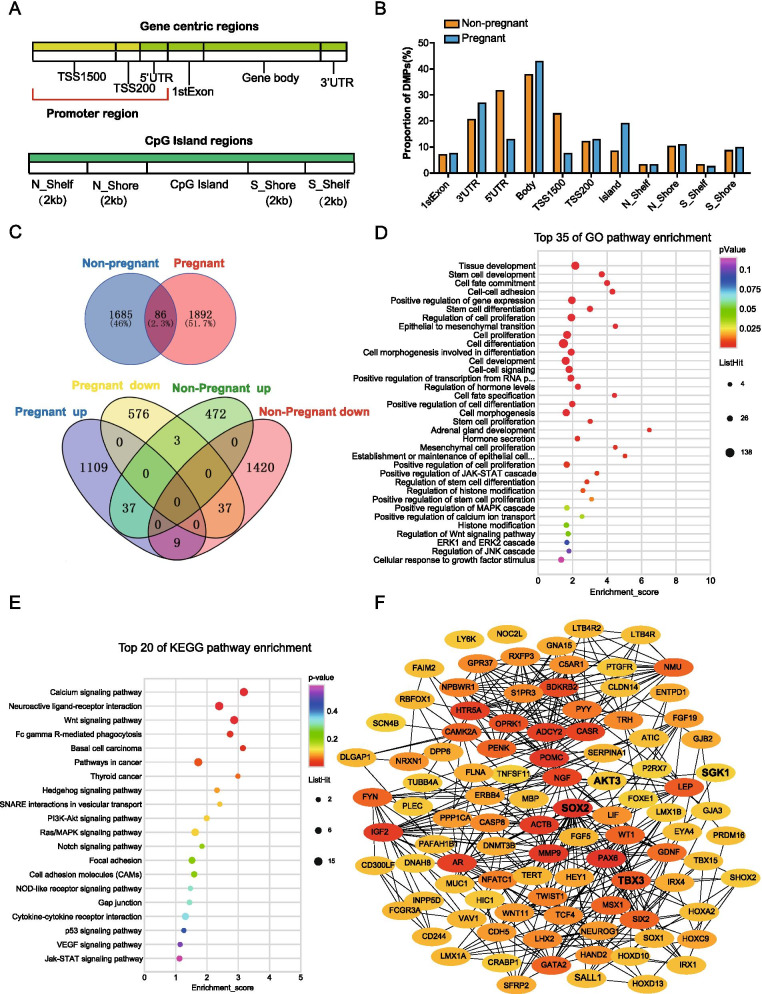
DNA methylation analysis of the promoter region between non-pregnant and pregnant groups. **A** Schematic diagram of gene regions and CpG island region. **B** Bar graph showing DNA methylation in different regions between the non-pregnant and pregnant groups. **C** Venn diagram showing the overlap and the number of differentially methylated of the promoter region DMPs between the non-pregnant and pregnant adrenocortical adenoma patients. **D**, **E** Enriched GO and KEGG pathways (Top 30) within just the pregnant promoter hypomethylated DMPs associated genes (*P* < 0.05). **F** PPI and functional enrichment network of just pregnant promoter hypomethylated DMPs associated genes using the STRING and Cytoscape software

The DNA promoter region is important for gene expression regulation. Hypomethylation is typically used as a positive gene expression regulator. We compared the DNA promoter region methylation status between the two groups (Fig. 5A, B). In general, the pregnant group had a lower DNA promoter region methylation percentage than non-pregnant group (33.25% vs 66.48%). Similar results with the genome-wide DNA methylation pattern, the DNA promoter region indicated that there were 1978 methylated probes in the pregnant group, among them 576 DMPs were completely hypomethylated and 1109 DMPs were completely hypermethylated (Fig. 5C).

The GO and KEGG pathway analysis were used to investigate the DNA promoter region epigenetic status of the pregnant group. The GO enrichment analysis showed that hypomethylated 488 genes and most genes were significantly enriched in tissue development, stem cell development, cell fate commitment, cell–cell adhesion, cell proliferation, cell development, stem cell proliferation, and cell–cell signaling (Fig. 5D and Additional file 5). We also conducted the KEGG Enrichment analysis on 488 genes. We discovered some genes enriched linked to tumor proliferation signaling pathway, including 13 genes that were associated with Wnt signaling pathway, 10 genes with Ras/MAPK signaling pathway, 4 genes with PI3K-Akt signaling pathway,2 genes with p53 signaling pathway, 2 genes with VEGF signaling pathway and 4 genes with Jak-STAT signaling pathway (Fig. 5E and Additional file 6).

We then used STRING-database and Cytoscape software to analyze the 488 genes. It displays 432 nodes and shows 91 edges, then we analyzed the functional enrichment in the network, focusing on the key proteins that regulate cell population proliferation, cell and cell adhesion, cell death, and mitotic cell cycle. It was considered that those top genes with high degree of connectivity as the hub genes of ACC: SOX2 (SRY (sex determining region Y)-box 2), PAX6 (Paired Box 6), POMC (Pro-opiomelanocortin), MMP9 (Matrix metallopeptidase 9), IGF2 (Insulin Like Growth Factor 2), AR (Androgen Receptor), ADCY2 (Adenylate Cyclase 2), HTR5A (5-Hydroxytryptamine Receptor 5A), NGF (Nerve growth factor), CASR (Calcium Sensing Receptor), BDKRB2 (Bradykinin Receptor B2), OPRK1 (Opioid Receptor Kappa 1), AKT3 (AKT Serine/Threonine Kinase 3), SGK1 (Serum/Glucocorticoid Regulated Kinase 1), TBX3 (T-Box Transcription Factor 3) (Fig. 5F and Additional file 7).

### Identification and validation of the promoter region DNA methylation marker

**Fig. 6:**
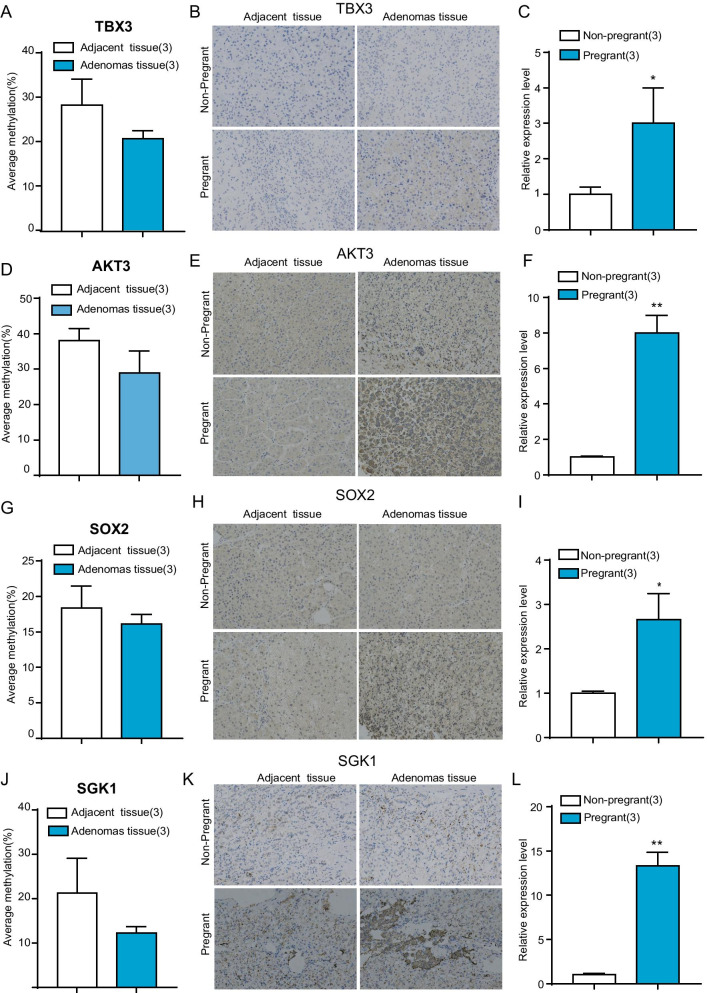
Identification and validation of the promoter region DNA methylation marker. **A**, **D**, **G**, **J** Percentages of TBX3 (**A**), AKT3 (**D**), SOX2 (**G**), SGK1 (**J**) DNA methylation level of adrenocortical adenoma tissue between non-pregnant and pregnant groups. **B**, **E**, **H**, **K** Immunohistochemical staining of adrenocortical adenoma tissue with anti-TBX3 (**B**), AKT3 (**E**), SOX2 (**H**), SGK1 (**K**) antibodies between non-pregnant and pregnant groups. **C**, **F**, **I**, **L** Statistical analysis of figure (**B**, **E**, **H**, **K**). Statistical significances are represented by asterisks *P < 0.05; **P < 0.01

The above selected four genes have been described as potential oncogenes in hypopharyngeal carcinoma [10], breast cancer [11], osteosarcoma [12], and adrenocortical adenomas [13]. Bisulfite pyrosequencing was used to verify their promoter methylation profiles. The promoter region DNA average methylation was TBX3 (28.20% versus 20.61%), AKT3 (38.90% versus 28.90%), SOX2 (18.38% versus 16.14%), and SGK1 (21.33% versus 12.33%) in the two groups (Fig. 6A, D, G, J).

To determine whether the promoter hypomethylation was linked to the higher protein expression of the above four genes. we performed immunohistochemistry to examine the degree of protein expression and found that hypomethylation of the above four genes were linked to a higher protein staining level (Fig. 6B, E, H, K), Statistical analysis also showed that the pregnant group had higher levels of TBX3, AKT3, SOX2, and SGK1 than non-pregnant adenomas group, with a significant difference (*P* < 0.05 or *P* < 0.01) (Fig. 6C, F, I, L).

## Discussion

In all our patients, CS was caused by CPA. Considering the tumor size in our four pregnant patients, the adrenal mass could have been preexisting before pregnancy. Thus, their ovulatory function was not affected, whether accompanied by subtle hypercortisolism. In the present study, a significant shorter duration, with no difference in tumor size, was observed in pregnant patients with CPA compared with non-pregnant patients, which indicated a faster growth rate of adenoma in patients during pregnancy. This was also proved by the higher expression of Ki-67, a good marker of proliferation, in adrenocortical adenoma in the pregnant group. Therefore, we hypothesized that hormones and internal environment during pregnancy affected the expression of genes associated with the proliferation of adrenocortical adenoma.

HCG is one of the most important pregnancy-related hormones and rises rapidly during early pregnancy. hCG upregulated LHCGR, GATA4, and ZFMP2 expression and induced hCG-responsive cells to differentiate into adrenal steroid-producing cells [8]. Furthermore, cortisol hypersecretion was found after administration of hCG and luteinizing hormone-releasing hormone (LHRH) [14]. However, we did not observe cortisol hypersecretion in all patients during pregnancy or after menopause. Indeed, hCG administration did not stimulate cortisol secretion in pre- or post-menopausal women [15]. Similarly, some cases with adrenal tumors or macronodular which express LHCGR mRNA but did not show cortisol response to hCG, LH, or GnRH [16, 17]. All investigations suggested that an alternative pathogenesis existed in the CPA during pregnancy.

In recent years, epigenetic modification has been proved to play important roles in regulating gene expression, among which DNA methylation is the main fundamental epigenetic mechanism [18]. Importantly, depending on the underlying genetic sequence, DNA methylation in different genomic regions may have different effects on gene activity. Within intergenic regions, one of the main roles of DNA methylation is to repress the expression of potentially harmful genetic elements [19]. Research shows that DNA methylation has been found participating in the pathogenesis of adrenocortical hormone-producing adenoma. DNA methylation plays a regulatory role for CYP11B2 expression and might contribute to aldosterone hypersecretion in aldosterone-producing adenoma (APA) [20]. DNA hypomethylation at the CYP11B1 promoter upregulated the expression of CYP11B1 and promoted cortisol production in CPA [4]. Besides, DNA hypomethylation of synaptophysin (SYP) promoter increased the expression level of SYP, promoting cortisol secretion and proliferation of CPA [21]. Several studies had demonstrated that some of the pregnancy-related factors can influence maternal DNA methylation, which was closely associated with gestational diabetes and anemia, adverse pregnancy outcomes like preterm birth, and preeclampsia [22, 23]. Therefore, we hypothesized that DNA methylation may be involved in pregnant patients with CPA through regulating the expression of genes associated with the progression of adrenocortical adenoma.

In the present study, our results indicated that the CPAs were exacerbated during pregnancy. We hypothesized that changes in hormones and internal environment during pregnancy promotes tumor progression through the hypomethylation promoter of proliferation-related genes. 488 genes of the pregnant group that hypomethylated in the promoter sites were analyzed using bioinformatics. We found that these genes were mostly involved in the processes of cell proliferation, adhesion, and death. After reviewing the literature on the 488 genes mentioned above and combining the results of the KEGG pathway and functional enrichment network, four genes, including TBX3, AKT3, SOX2, and SGK1, involved in cell proliferation were detected.

These four genes are part of the WNT, MAPK, and PI3K signaling pathways, which play important roles in the proliferation of adrenocortical adenomas [24–26]. Pyrosequencing was performed to verify the DNA methylation of the above four genes. Our results showed that the promoter regions of the above-mentioned genes also had low methylation levels in the CPA tumor tissues during pregnancy compared with the adjacent tissues, which is consistent with the results of Illumina's Infinium human methylation 850 K BeadChip. Although the statistical analysis of pyrophosphate showed no significant difference, we considered that it was caused by the relatively small number of samples. Furthermore, we stained the tissue samples with HE and found that the four gene expression significantly increased along with hypomethylation of promoter.

Meanwhile, our research still has some limitations. First, only four samples were obtained because of the rarity of CS during pregnancy. Second, proteomics and transcriptomics research for verification the gene expression status and function were not performed without no fresh samples. Third, further studies are needed to verify the gene functions in the mechanism of CPA during pregnancy by using animal and cell experiments.

## Conclusion

In the present study, our results showed that CPA grow rapidly during pregnancy. Through analyzing the genome-wide DNA methylation profiling of CPA in non-pregnant and pregnant groups, our study indicated that alterations of DNA methylation were associated with the pathogenesis and exacerbation of CPA during pregnancy. We firstly provide a DNA methylation database about Cushing’s syndrome during pregnancy and make these data available to the public. Further studies are needed to investigate the specific function of DNA methylation in the development of CPA during pregnancy.

## Methods

### Patients

Four pregnant and twelve non-pregnant patients of childbearing age with CPA were enrolled form Qilu Hospital of Shandong University form January 2019 to June 2020. Surgically resected CPA and paired adjacent specimens from all participants were obtained. Comprehensive clinical data such as physical examination, medical history, biochemical detection, and imagological examination were collected. All procedures involving human participants were carried out in accordance with the institutional and/or national research committee's ethical standards, as well as the 1964 Helsinki Declaration and its subsequent amendments or comparable ethical standards. This study was also approved by the ethics committee of Qilu Hospital of Shandong University (No: KYLL-2020(KS)-083). All subjects in this study signed a consent form voluntarily, with the ethical committee's approval.

### Data collection and measurements

Basic demographic information was collected by the data collection system of Qilu Hospital, including age, BMI, blood pressure (BP), and duration of disease. The time of disease onset was identified as the presence of typical signs of hypercortisolism, such moon-shaped face and purple striae. After fasting for at least 10 h, venous blood was collected for the measurement of potassium, follicle-stimulating hormone (FSH), LH; estradiol (E2), prolactin (PRL), progesterone (P) and HCG. Cortisol rhythm (8 am, 4 pm, 12 pm) was also conducted for each patient. Some patients also finished the 24 h urinary free cortisol examination. All patients completed the adrenal CT examination. Tumor size (cm^2^) was calculated based on the longest and wide of surgically resected CPA. Growth rate of the tumor was calculated as tumor size (cm^2^) divided by the duration of the disease (months).

### Diagnosis, treatment, and clinical outcome of CPA

The diagnostic criteria of CPA in this study were as follows: (1) high level of cortisol and low level of ACTH; (2) abnormal cortisol rhythm; (3) adrenal occupying lesion confirmed by adrenal CT image; (4) tumor was confirmed in the adrenal cortex by pathology. All patients underwent adrenalectomy. For four pregnant patients, one patient had placental abruption, and two patients occurred stillbirth.

### Preparation of paraffin samples

Paraffin samples from 4 non-pregnant and 4 pregnant patients were sent for hematoxylin/eosin (HE) staining and 850 K BeadChip analysis. One sample of pregnant patient could not meet the testing requirements. Therefore, 3 samples of non-pregnant and pregnant patient were used for further bisulfite pyrosequencing, respectively.

### Tissue microarray construction

For 4 non-pregnant and 3 pregnant patients with CPA, 6 μm hematoxylin/eosin (HE)-stained sections of benign and malignant adrenocortical tissue were reviewed to confirm the diagnosis and target areas of the block for tissue microarray construction.0.6-mm-diameter core biopsies were taken using a precision instrument (Beecher Instruments, Silver Spring, MD) as described [13]. On a recipient paraffin block, the tissue cores were arrayed in triplicate. Tissue array blocks were cut into 6 m sections and placed on charged poly-lysine–coated slides.

### DNA isolation and Infinium human methylation 850 K BeadChip

Infinium Human Methylation 850 K BeadChips (Illumina) were used to determine methylation levels across the genome. The chip contains 91% of the original 450 K chip sites, plus an additional 413,745 sites (a total of 866,895 CpG sites). Following bisulfite treatment with the EZ DNA Methylation Kit (Zymo Research) according to the manufacturer's instructions, the processed DNA samples were hybridized to the Illumina BeadChip using the Illumina Infinium HD Methylation Protocol. The detection was finished by Shanghai Whale Boat Gene Technology Co.

### Gene ontology and KEGG pathway enrichment analysis of differently methylated genes

We annotated the two groups differently methylated genes using the Gene Ontology (GO) and Kyoto Encyclopedia of Genes and Genomes (KEGG) databases to clarify the biological functions and the signaling pathways involved. Fisher's exact test was used to calculate the enrichment.

### The establishment of a PPI network and the selection of hub modules analysis

To assess protein–protein interactions, the Search Tool for the Retrieval of Interacting Genes (STRING) database (http://www.string-db.org/) was used (PPI). Furthermore, the database was used to quantify the relationships between the different methylated genes. Then, we built a PPI network with Cytoscape software (3.8.2) containing the Cytohubba software. The genes with the highest node score and connectivity were chosen.

### Bisulfite pyrosequencing method validation

Bisulfite pyrosequencing was used to validate the 850 K array. The selected promoter sites were cg06542648, cg13307058, cg20106776 and cg15323015, cg06542648 located in the *Tbx3* gene, cg13307058 located in the *Sgk1* gene, cg20106776 located in the *Sox2* gene and cg15323015 located in the *Akt3* gene, We used genomic DNA extraction kit (QIAGEN DNeasy kit, Cat: 69,506) for DNA extraction. The Qiagen EpiTect Bisulfite Kit (Qiagen, Cat: 59,104) was used for detecting methylation. PyroMark Assay Design 2.0 was used for primer design. The primer sequence information was shown in Table 2. The PCR amplification system was total 50 μL reagent volume including 34.8 μL H_2_O, 10 μL 5× buffer GC (KAPA), 1 μL dNTP (10 mM/each), 1 μL Primer (up50pM/ul), 1 μL Primer (down50pM/ul) and 0.2 μL Template 2 Taq (5U/μL). The PCR amplification procedure was used as follows: 40 cycles, 95℃ 3 min, 94℃ 30 s, 56℃ 30 s, 72℃ 1 min, 72℃ 7 min, 4℃ forever.

**Table 2:** The primers sequence information for Bisulfite Pyrosequencing

Primers name	Primer sequence (5′–3′)	5′ modification
CG06542648-F(242 bp)	AGAATTAAGAATTGTGGGTTTAGG	
CG06542648-R(Q48)	CCACCCCCAAAACTTAATCTAACCAAAA	5′-Biotin
CG06542648-S	GGGAGTTGAGGTAATAGAG	
CG13307058-F(104 bp)	GTAGAAGGTAGGGAAGAGAGG	
CG13307058-R(Q96)	TCCCAAACATCCCCCAAC	5′-Biotin
CG13307058-S	GGAAGAGAGGGAAGT	
CG20106776-F(175 bp)	AGAGTTTTTAGGATTTTAGTAGAATTAGT	
CG20106776-R(Q96)	TCTCCCAATATACCCTACCTACACCTATAC	5′-Biotin
CG20106776-S	AGATTTTTATTGTGGTGG	
CG15323015-F(151 bp)	ATTAGAGAGTGGGAAGGGTAGT	
CG15323015-R(Q96)	TTACCCCTCTTCTAAACCCAACCTT	5′-Biotin
CG15323015-S	AGGGGAGTTATTATGAG	

### Immunohistochemical and molecular analysis

Sections from adrenocortical adenomas and adjacent tissue arrays were deparaffinized, rehydrated in various alcohol grades, and then microwave oven treated (15 min in 0.01 M-citrate buffer at pH 6.0). The tissue sections were reacted with anti-TBX3 (Santa Cruz, Sc-166623, dilution 1:50; USA), anti-SGK1 (Santa Cruz, sc-28338, dilution 1:50; USA), anti-AKT3 (Proteintech, 21641-1-AP, dilution 1:50; China) or anti-SOX2 (Proteintech, 11064-1-AP, dilution 1:50; Sigma–Aldrich) antibodies overnight at 4 °C. Immunohistochemical staining was done as previously described [7]. Immunofluorescence staining was performed for Ki-67 (diluted 1:100; GB111141; Servicebio, Wuhan, China), DAPI (diluted 1:5000, C1002, Beyotime). Immunofluorescence staining was done as previously described [27].

### Statistical analysis

Data were displayed as median (min–max), mean SEM, or percentages, depending on the circumstances. The student's *t* test and Fisher's exact test were used to compare the clinical presentation, stage of the tumor at discovery, and hormonal secretion between pregnant and nonpregnant patients. Wilcoxon rank sum paired test for normalized values of the two groups was used to identify consistently differentially whole and CpG methylated sites. The data analysis was performed using the GraphPad Prism 8 software. As a significant difference, *P* < 0.05 was used.

## Supplementary Information

**Additional file 1: **Adrenocortical carcinoma associated methylation genes that have been reported previously.

**Additional file 2: **Enriched GO pathways (Total) in hypo-DMPs associated with DNA hypomethylation genes in the adenoma tissue of the non-pregnant patients.

**Additional file 3: **Enriched KEGG pathways (Total) in hypo-DMPs associated with DNA hypomethylation genes in the adenoma tissue of the non-pregnant patients.

**Additional file 4: **The detail interaction results of the overall region hypomethylated DMPs associated genes in the adenoma tissue of the non-pregnant patients using the STRING software.

**Additional file 5: **Enriched GO pathways (Total) within just the pregnant promoter hypomethylated DMPs associated genes.

**Additional file 6: **Enriched KEGG pathways (Total 30) within just the pregnant promoter hypomethylated DMPs associated genes.

**Additional file 7: **The detail interaction results of just pregnant promoter hypomethylated DMPs associated genes using the STRING software.

## Data Availability

All relevant data and material are available from the corresponding author upon request.

## Abbreviations

CS: Cushing’s syndrome
CPA: Cortisol-producing adrenocortical adenoma
PPI: Protein–protein interaction
ACTH: Adrenocorticotropic hormone
LH: Luteinizing hormone
HCG: Human chorionic gonadotropin
CYP11B1: Cytochrome P450 family 11 subfamily B number 1
FSH: Follicle-stimulating hormone
E2: Estradiol
PRL: Prolactin
P: Progesterone
KEGG: Kyoto Encyclopedia of Genes and Genomes
LHRH: Luteinizing hormone-releasing hormone
SYP: Synaptophysin
