# Supplementary material for: Alterations of DNA methylation were associated with the rapid growth of cortisol-producing adrenocortical adenoma during pregnancy
Source: Clin Epigenetics. 2021 Dec 4;13:213. doi: 10.1186/s13148-021-01205-3 (PMC8642905; doi:10.1186/s13148-021-01205-3)
Supplement: Supplementary file 1 — Additional file 1: Adrenocortical carcinoma associated methylation genes that have been reported previously. [file 13148_2021_1205_MOESM1_ESM.doc]

**Additional file 1. Adrenocortical carcinoma associated methylation genes that have been reported previously.**

| **Study** | **Country** | **Year** | **Number** | **Method** | **Hypomethylated** | **Hypermethylated** |
| --- | --- | --- | --- | --- | --- | --- |
| [12] | USA | 2016 | 24 | Infinium HumanMethylation 450 BeadChips (Illumina, San Diego, CA) | ADCY2  TMEM132D  TP53  SHANK1  TWIST2 | CCDC8 |
| [5] | USA | 2016 | 91 | The Illumina Infinium HM450 array | TERT  TP53  AIG1  CASP1  IGF2  NHS  SOX17 |  |
| [6] | USA | 2012 | 87 | Infinium HumanMethylation 450 BeadChips (Illumina, San Diego, CA, USA) | LPAR2  TRT3 | CD55  MEIS2  KCNQ1 |
| [7] | France | 2015 | 135 | Infinium HumanMethylation27 Beadchip (Illumina, San Diego, CA) MS-MLPA |  | KRT8 |
| [13] |  | 2012 | 27 | HumanMethylation27 BeadChip | GATA4 |  |
| [14] | Michigan. | 2019 | 55 | Infinium HumanMethylation450 BeadChip (“450k”) platform | CDK6 |  |
| [15] | USA | 2019 | 48 | human Infinium MethylationEPIC BeadChip array (Illumina, San Diego, CA) | GNAS |  |
